# What the London Hospitals Are Doing

**Published:** 1914-11-07

**Authors:** 


### HOSPITALS AND THE WAR.

### What the London Hospitals are Doing.

THE influx of wounded, which necessarily continues to arrive, is keeping the London hospitals very busy, and we append below a summary of the conditions obtaining at a few typical institutions:—

At St. Bartholomew's Hospital one wing, containing 174 beds, was placed at the disposal of the War Office early in August, but it so happened that it was not until October 14 that the accommodation was made use of. On that and the following date two batches of Belgian wounded, totalling 130, arrived, and at the end of ten days one-third of their number had been discharged. On October 25 sixty-one British wounded arrived, making the total of British and Belgian in the hospital 142. Seventeen of the latter were discharged by October 28, when forty more British were admitted, which made the highest number yet installed in the reserved wing—namely, 160. Up to Wednesday this week further discharges had reduced the number to 121. In other words, the wing set apart for the wounded has been about two-thirds occupied since the first demand upon it was made in the middle of October.

The position at the Middlesex Hospital has been as follows:—Beds to the number of eighty have been set apart for the wounded in the hospital, while 130 have been reserved at the convalescent home at Clacton-on-Sea. So far, these have been kept occupied by arrivals following on discharges, and, as was anticipated, a further batch of twenty was admitted to the hospital on Tuesday last.

At the Royal Free Hospital, which has been appointed as a section of the 4th London General Hospital, forty casualties have been received, and at the time of writing a further batch is expected. In order to carry out this work it has been necessary, here as elsewhere, somewhat to increase the number of beds in the wards. The consideration which determined this course was also that such procedure would dislocate as little as possible the ordinary work of the hospital in treating the poor of the neighbouring districts.

At North London, or University College Hospital, three wards have been set aside for the wounded. At the present time there are 100 wounded soldiers in the institution, but the reserved wards have been able to accommodate them, except that a few extra beds have been placed in each ward. In other respects no special arrangements have been necessitated.

There are no wounded at St. Mary's Hospital. At Guy's thirty wounded officers have been received this week, and are being accommodated in a special ward, which is fitted with cubicles.

There are at present sixty-eight wounded soldiers in Westminster Hospital, of whom fifty-one are Belgians and seventeen English and Scottish. The Belgians arrived first, and had been engaged on the Nieuport-Dixmude front. Some of them are severely injured, and one of them died within twenty-four hours of admission. It is universally recognised that they are a charming people. The English and Scottish soldiers belong to fourteen different regiments, one of them being a member of the London Scottish. All had been fighting in the neighbourhood of Ypres, and, fortunately, none of them are very severely wounded, their injuries for the most part being shrapnel and bullet wounds in the extremities. They describe the fighting at Mons as child's play compared with that they have lately been engaged in.

